# Species classification of *Tabanus* (Diptera: Tabanidae) in Western Thailand: Integrating DNA barcoding and modern morphometrics

**DOI:** 10.1016/j.crpvbd.2025.100243

**Published:** 2025-01-13

**Authors:** Tanasak Changbunjong, Thekhawet Weluwanarak, Sedthapong Laojun, Tanawat Chaiphongpachara

**Affiliations:** a Department of Pre-Clinic and Applied Animal Science, Faculty of Veterinary Science, Mahidol University, Nakhon Pathom, 73170, Thailand; b The Monitoring and Surveillance Center for Zoonotic Diseases in Wildlife and Exotic Animals (MoZWE), Faculty of Veterinary Science, Mahidol University, Nakhon Pathom, 73170, Thailand; c Department of Public Health and Health Promotion, College of Allied Health Sciences, Suan Sunandha Rajabhat University, Samut Songkhram, 75000, Thailand

**Keywords:** Horse flies, Species identification, DNA barcode, Geometric morphometrics, Landmark-based geometric morphometrics, Outline-based geometric morphometrics

## Abstract

The species of *Tabanus*, commonly known as horse flies, are remarkable ectoparasites capable of transmitting various pathogens to animals and humans. Given their role in disease transmission, accurate identification of horse fly species is critical but traditionally relies on morphological characteristics, requiring significant expertise and posing a high potential for error, especially with damaged specimens. To address the limitations of traditional morphological identification, this study highlights the importance of alternative techniques, including DNA barcoding and geometric morphometrics (GM). To enhance the reliability of species identification, DNA barcoding was employed to analyze 30 cytochrome *c* oxidase subunit 1 (*cox*1) gene sequences from 15 horse fly species, which were then compared with sequences in the GenBank and BOLD databases. Most *cox*1 sequences aligned with existing data, with similarity percentages ranging from 96% to 100%. However, discrepancies were noted, including *Tabanus helvinus*, misidentified as *Tabanus aurilineatus*, and *Tabanus minimus*, whose sequences matched those of both *Tabanus minimus* and *Tabanus mesogaeus*. Besides DNA barcoding, GM analyses were conducted to enhance species classification accuracy. Our GM analyses employed the landmark-based method for the entire wing and the outline-based method for the first submarginal cell. While shape-based GM analyses demonstrated high reliability, with adjusted total accuracy scores of 97% and 96%, size-based GM analyses yielded significantly lower accuracy, with scores of only 27% and 23%, respectively. These findings provide a foundation for refining horse fly species classification by integrating DNA barcoding and GM approaches, offering valuable advances in species identification and developing targeted control measures.

## Highlights

• DNA barcoding and geometric morphometrics improve *Tabanus* species classification accuracy.
• *cox*1 gene sequences show 96–100% match but reveal misidentifications in databases.
• Wing shape and size analyses have high and low classification accuracy, respectively.

## Graphical abstract

**Figure:**
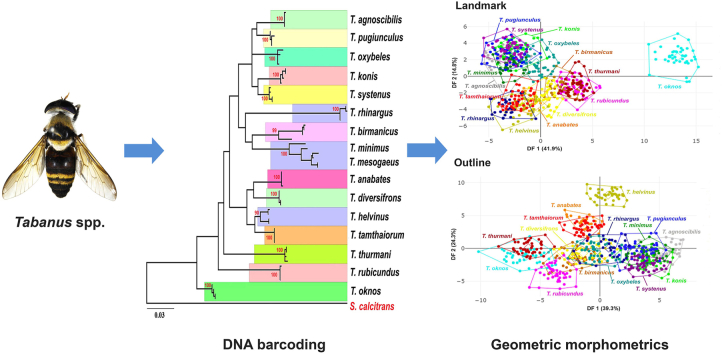

## 1 Introduction

The species of *Tabanus*, commonly known as horse flies, belong to the family Tabanidae (Diptera) which comprises approximately 4665 species globally, distributed across 177 genera, with 1386 species classified under the genus *Tabanus* (Evenhuis and Pape, 2024). Driven by their biological needs, adult female horse flies require blood meals from domestic or wild animals to develop their eggs (Mullens, 2019). Occasionally, they have also been reported to attack humans, particularly in areas with few animals (Mullens, 2019). Recognized as mechanical vectors, horse flies can transmit a wide range of pathogens, including protozoans, bacteria, and viruses, making them significant vectors of diseases that impact both veterinary and human health (Krinsky, 1976; Foil, 1989; Baldacchino et al., 2014; Mullens, 2019; Lendzele et al., 2022). Furthermore, their relatively large size (10–30 mm) and painful, persistent biting behavior make them a considerable nuisance to people and livestock, adversely affecting outdoor activities, tourism, and agriculture (Baldacchino et al., 2014). This global relevance is mirrored in Thailand, where the tropical climate supports a rich diversity of disease vectors, including horse flies.

Thailand, a Southeast Asian country with a tropical climate, harbors a rich diversity of disease vectors, including horse flies (Burton, 1978; Tumrasvin, 1989; Changbunjong et al., 2018b). Approximately 80 horse fly species have been identified across the country (Burton, 1978). The western region, particularly Kanchanaburi Province, hosts the highest diversity, with 32 identified species (Changbunjong et al., 2018b). This diversity is largely attributed to the region’s extensive rainforests, which provide abundant green spaces (Changbunjong et al., 2018b). These forests, characterized by mountainous and hilly terrains covered with a mix of deciduous and tropical vegetation, are frequently used by villagers for grazing livestock, particularly cattle and buffalo. These animals serve as crucial feeding sources for horse flies (Changbunjong et al., 2018b). Given the high diversity of horse flies in regions like Western Thailand, accurate species identification becomes a critical challenge.

Traditionally, horse fly species identification relies on morphological characteristics to differentiate between species (Burton, 1978; Mugasa et al., 2018). Key features used for identification include head structures, traits of the callus, antennae, eyes, frons, and beard, along with the coloration and patterns of the body, legs, and wings (Burton, 1978). While this method is straightforward and cost-effective, as it does not require advanced scientific equipment, it comes with significant limitations. Accurate identification demands substantial expertise to discern subtle differences, and the potential for error is high, particularly when specimens are damaged during field collection or by natural causes. Misidentifications not only lead to misinformation but also hinder effective disease control, as they result in inaccurate horse fly population management (Changbunjong et al., 2018a; Rodrigues et al., 2024). To address these challenges, integration of modern techniques such as DNA barcoding and geometric morphometrics (GM) into the classification process may ensure accurate identification of these disease vectors. To overcome the limitations of morphological methods, molecular techniques such as DNA barcoding have emerged as powerful tools for species identification.

The DNA barcode method has become a widely accepted and highly accurate molecular approach for identifying animal species, including insect disease vectors such as mosquitoes (Chaiphongpachara et al., 2022; Laojun et al., 2023b), deer flies (Changbunjong et al., 2020b), sand flies (Nzelu et al., 2015), black flies (Pramual et al., 2021), and stomoxyine flies (Changbunjong et al., 2016). Recently, DNA barcoding has been successfully applied to identifying horse fly species in Thailand (Changbunjong et al., 2018a) and has facilitated the discovery of new fly species in the subfamily Stomoxyinae, such as *Haematobosca aberrans*, in Thailand (Changbunjong et al., 2020a). While DNA barcoding excels in resolving taxonomic ambiguities, GM provides complementary methods by analyzing morphological traits precisely.

Geometric morphometrics, particularly through wing analyses, is increasingly used to identify medically and veterinary-important insects with similar morphologies (Laojun et al., 2023a, 2023b; Jeon et al., 2024). GM offers several advantages, it is fast, cost-effective, and straightforward. While digitizing the coordinates of landmarks requires some knowledge of the organism, it does not demand extensive technical expertise (Lorenz et al., 2017). This technique has also proven effective in distinguishing certain species of horse flies, such as *Tabanus megalops*, *Tabanus rubidus*, *Tabanus striatus*, *Tabanus triangulum*, and *Tabanus occidentalis*, which have been successfully identified using GM techniques (Changbunjong et al., 2021; Rodrigues et al., 2024). Among the GM methods, the landmark-based approach uses coordinates of anatomical landmarks to analyze both size and shape, facilitating comparisons across populations and species (Dujardin, 2008). This method is widely favored for vector species identification due to its simplicity, requiring only a few coordinate points for analysis. In contrast, the outline-based GM method employs pseudo-landmarks to define contours or boundary outlines rather than true anatomical landmarks. While this method is less popular due to the complexity of constructing outlines with numerous pseudo-landmarks (Dujardin et al., 2014; Laojun et al., 2024), recent studies have demonstrated its utility. The contours of wing cells, particularly the first submarginal cell, exhibit unique species-specific traits in horse flies, making the outline-based GM method essential for their classification (Changbunjong et al., 2024a). Consequently, both landmark- and outline-based methods should be tested on a broader range of horse fly species to validate these findings. To further enhance classification accuracy and precision, GM techniques should be complemented by molecular methods, such as DNA barcoding. Building on these methodologies, this study aims to evaluate their effectiveness for classifying horse fly species in Western Thailand, a hotspot of species diversity.

To evaluate the effectiveness of these alternative techniques for classifying multiple horse fly species in Western Thailand - a region with the highest diversity of these species - both landmark- and outline-based GM methods were employed. DNA barcoding was also used to verify the accuracy of morphological classification, ensuring sample correctness. The findings from this research provide practical guidelines for classifying horse fly species using various alternative methods, offering valuable insights for future practitioners and researchers.

## 2 Materials and methods

### 2.1 Study sites

**Fig. 1:**
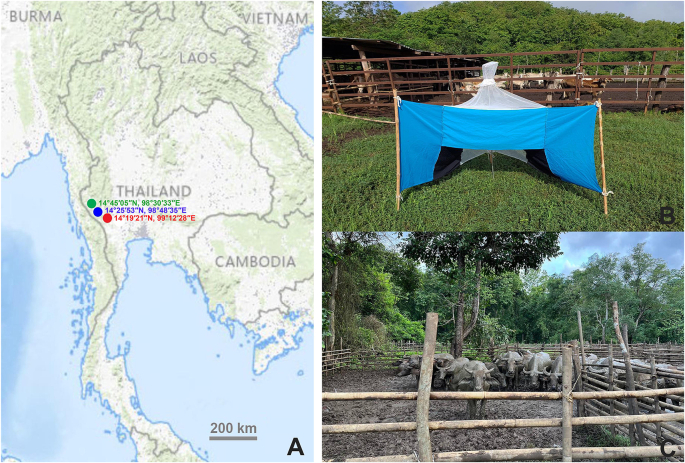
**A** Collection sites in Kanchanaburi, Western Thailand: Si Sawat District (*red*), Sai Yok District (*blue*), and Thong Pha Phum District (*green*). **B** An Nzi Trap used to capture horse flies. **C** Buffalo, common hosts for horse flies in the study areas. Map source: USGS National Map Viewer (https://apps.nationalmap.gov/viewer/).

Horse flies were collected from three districts in Kanchanaburi Province, Western Thailand: Si Sawat District (14°19′21″N, 99°12′28″E), Sai Yok District (14°25′53″N, 98°48′35″E), and Thong Pha Phum District (14°45′05″N, 98°30′33″E) (Fig. 1A). These locations were selected based on prior studies that identified them as areas with the highest species diversity of horse flies in Western Thailand (Changbunjong et al., 2018b). Each site features forested environments rich in flora and fauna, with dense vegetation creating suitable habitats. The landscape includes scattered mountains and rivers, contributing to ecological diversity. These areas also host small rural villages with low human populations. Buffalo and cattle, often brought to graze in these regions, are a common sight.

### 2.2 Horse fly collection and morphological identification

Nzi traps were employed for horse fly collection in the field monthly from March to July 2024 (Fig. 1B). The traps were locally made using blue and black Solon® fabric (100% polyester), sourced from Bangkok, Thailand. At each site, five traps were set up approximately 50 m apart, positioned near local villagers’ buffalo and cattle pens (Fig. 1C). Traps operated daily from 6:00 to 18:00 h over four consecutive days per month. Specimens were removed from the traps every 2–3 h, euthanized in a freezer (−10 °C), and stored in individual 1.5-ml microcentrifuge tubes. After the collection period at each site concluded, all specimens were transported by car to the Vector-Borne Diseases Research Unit, Faculty of Veterinary Science, Mahidol University.

Subsequently, only female horse flies were identified morphologically under a stereomicroscope using Burton’s standard taxonomic keys (Burton, 1978). Identified specimens were stored individually in 1.5-ml microcentrifuge tubes containing 95% ethanol and kept at −20 °C until DNA extraction was performed to confirm species identification.

### 2.3 DNA extraction, polymerase chain reaction and sequencing

DNA barcoding was employed to verify the species of horse flies before classification analysis *via* the GM technique. Two specimens per species were randomly selected for analysis. Under a stereomicroscope, two to three legs were detached from each specimen’s thorax. DNA was extracted using the DNeasy Blood and Tissue Kit (Qiagen, Hilden, Germany) following the manufacturer’s instructions. The barcoding primers LepF1 (5′-ATT CAA CCA ATC ATA AAG ATA TTG G-3′) and LepR1 (5′-TAA ACT TCT GGA TGT CCA AAA AAT CA-3′) (Hebert et al., 2004) were used to amplify a 658-base-pair (bp) fragment of the mitochondrial cytochrome *c* oxidase subunit 1 (*cox*1) gene. PCR amplification was conducted in a 25 μl reaction volume comprising 5 μl genomic DNA (approximately 50–100 ng), 0.1 μM of each primer, 1× PCR buffer, 1.5 mM MgCl_2_, 1.5 units of Platinum Taq DNA Polymerase (Invitrogen, Carlsbad, USA), 0.2 mM dNTPs, and distilled water to complete the volume. The cycling conditions included an initial denaturation at 94 °C for 1 min, followed by 5 cycles of 30 s at 94 °C, 40 s at 45 °C, and 1 min at 72 °C. This was followed by 35 cycles of 30 s at 94 °C, 40 s at 55 °C, and 1 min at 72 °C, ending with a final extension step at 72 °C for 10 min. To ensure accuracy, both positive and negative controls were included in each PCR round. Positive controls comprised DNA samples of *Haematobosca sanguinolenta* from prior studies (Changbunjong et al., 2024b) to verify equipment functionality, while distilled water was used as a negative control to detect PCR contamination.

To assess the quality of amplification, PCR products were separated by 1.5% agarose gel electrophoresis and stained with Midori Green DNA stain (Nippon Gene, Tokyo, Japan). Visualization was performed using an ImageQuant LAS 500 imager (GE Healthcare Japan Corporation, Tokyo, Japan) to confirm the expected DNA band sizes. Verified PCR products were then purified and sequenced bidirectionally through U2Bio (Thailand) Co., Ltd., Bangkok, Thailand.

### 2.4 Sequence analysis

The *cox*1 forward and reverse sequences were processed to generate consensus sequences, which were examined for deletions, insertions, and stop codons using BioEdit software version 7.2.5 (Hall, 1999). These consensus sequences were used for horse fly species identification by comparison with publicly available sequence data in the NCBI GenBank database through the Basic Local Alignment Search Tool (BLAST) (https://blast.ncbi.nlm.nih.gov/Blast.cgi) and the Barcode of Life Data Systems (BOLD) using the Barcode Identification Engine (https://id.boldsystems.org/). To ensure accuracy, the species identification process included verification of top-matching sequences in GenBank and BOLD to verify that multiple sequences supported the match. Once validated, 30 sequences of horse fly species were deposited in the GenBank database, under the accession numbers PQ462475-PQ462504 (see Supplementary Table S1 for details).

To analyze genetic relationships among taxa, maximum likelihood (ML) analysis was performed using MEGA software version 11 (Tamura et al., 2021). The substitution model was selected in MEGA based on the lowest Bayesian Information Criterion (BIC) value. The general time-reversible model with gamma-distributed rates plus invariant sites (GTR+G+I) was identified as the best-fit model for the sequence dataset. Nodal support was evaluated using bootstrap analysis with 1000 replicates. Sequences of horse fly species retrieved from GenBank (accession numbers in Supplementary Table S1) were included alongside sequences obtained in this study for validation. *Stomoxys calcitrans* (GenBank: KC960724), a member of the suborder Brachycera, was used as the outgroup. Finally, Figtree software version 1.4.4 was used to edit and visualize the phylogenetic analysis results (Rambaut, 2007).

### 2.5 Wing preparation for geometric morphometrics

Horse fly specimens with intact, undamaged wings were selected for GM analysis, with a total of 638 individuals included in the study. The left wings were dissected from the thorax, mounted on slides with Hoyer’s mounting medium, and examined under a stereomicroscope. The prepared slides were left to dry at room temperature for approximately one week before photographing the wings using a digital camera connected to the stereomicroscope. A total of 636 wing images were used for the landmark-based GM method, as two of the 638 images had unclear landmarks and were excluded. These images were also used for outline-based GM method; however, five images out of the 638 were excluded due to contour defects, leaving 633 images for analysis (Table 1). All procedures for both landmark- and outline-based GM analyses were conducted using XYOM (XY Online Morphometrics) version 3 (Dujardin and Dujardin, 2019), available at https://xyom.io/.

**Table 1:** Collection sites and number of wing images of *Tabanus* species used in landmark-based and outline-based geometric morphometric (GM) analyses.

Species	District/Province	Coordinates	No. of wing images used	
			Landmark-based GM analysis	Outline-based GM analysis
*T. agnoscibilis*	Si Sawat, Kanchanaburi	14°19′21″N, 99°12′28″E	45	43
*T. anabates*	Thong Pha Phum, Kanchanaburi	14°45′05″N, 98°30′33″E	25	24
*T. birmanicus*	Thong Pha Phum, Kanchanaburi	14°45′05″N, 98°30′33″E	45	45
*T. diversifrons*	Sai Yok, Kanchanaburi	14°25′53″N, 98°48′35″E	45	45
*T. helvinus*	Thong Pha Phum, Kanchanaburi	14°45′05″N, 98°30′33″E	45	45
*T. konis*	Si Sawat, Kanchanaburi	14°19′21″N, 99°12′28″E	45	45
*T. minimus*	Thong Pha Phum, Kanchanaburi	14°45′05″N, 98°30′33″E	45	45
*T. oknos*	Thong Pha Phum, Kanchanaburi	14°39′28″N, 98°32′19″E	43	44
*T. oxybeles*	ThongPha Phum, Kanchanaburi	14°39′28″N, 98°32′19″E	45	45
*T. pugiunculus*	Si Sawat, Kanchanaburi	14°19′21″N, 99°12′28″E	45	45
*T. rhinargus*	Thong Pha Phum, Kanchanaburi	14°39′28″N, 98°32′19″E	29	28
*T. rubicundus*	Sai Yok, Kanchanaburi	14°25′53″N, 98°48′35″E	45	44
*T. systenus*	Si Sawat, Kanchanaburi	14°19′21″N, 99°12′28″E	45	45
*T. tamthaiorum*	Thong Pha Phum, Kanchanaburi	14°39′28″N, 98°32′19″E	44	45
*T. thurmani*	Thong Pha Phum, Kanchanaburi	14°45′05″N, 98°30′33″E	45	45
Total			636	633

**Fig. 2:**
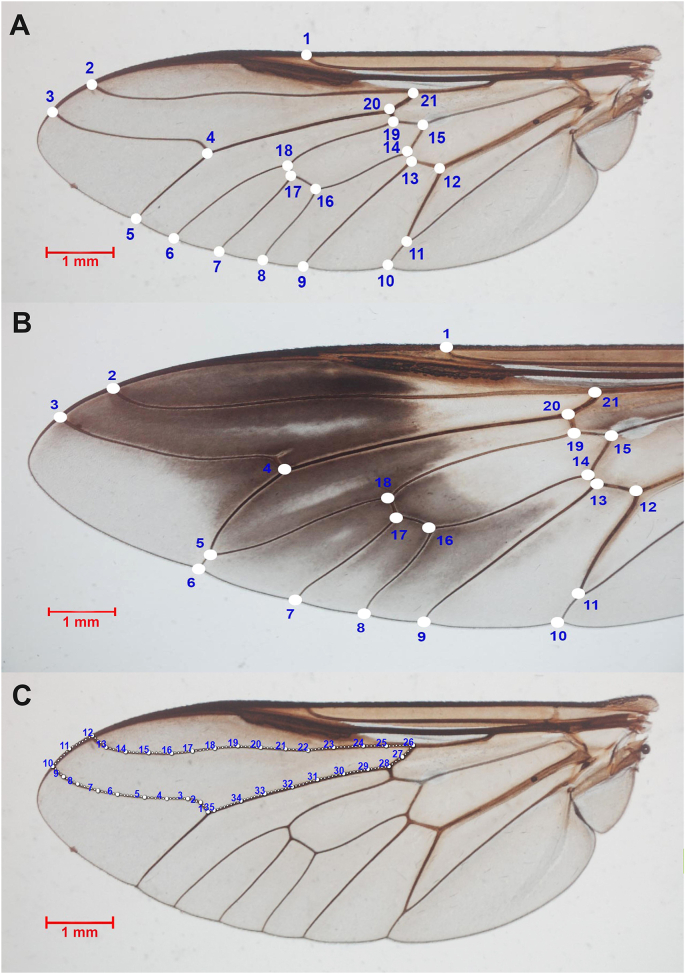
Female *Tabanus* wing configurations for geometric morphometric (GM) analyses. **A** Landmark locations for species with an open first posterior cell. **B** Landmark locations for species with the first posterior cell closed before the hind margin. **C** First submarginal cell contour used in outline-based GM analysis.

For the landmark-based GM method, 21 landmarks were selected and digitized on the wings, as illustrated in Fig. 2A and B. These landmarks were chosen based on clearly visible wing features to minimize ambiguity in coordinate determination, with slight adaptations from Changbunjong et al. (2021). Notably, variation in the pattern of landmark locations was observed among species. For example, in *Tabanus oknos*, the first posterior cell is closed before the hind margin (Fig. 2B), contrasting with other species where this cell remains open (Fig. 2A). For the outline-based GM method, the contour of the first submarginal cell of the wings was digitized (Fig. 2C). This wing cell has been shown in recent studies to be highly effective for distinguishing morphologically similar horse flies in Thailand (Changbunjong et al., 2024a). To ensure precision in the digitized landmark coordinates and the contour of the first submarginal cell, a repeatability test was conducted using the Procrustes ANOVA method (Klingenberg and McIntyre, 1998), in which 10 wing images from each species were randomly selected and digitized twice by the same operator. Additionally, to evaluate the influence of wing size on shape variation, the allometric effect was assessed by calculating the coefficient of determination (*r*^*2*^) from the regression of the first discriminant factor (DF) of shape against wing size.

### 2.6 Landmark-based GM analysis

Generalized Procrustes analysis was employed to standardize the landmark configurations by scaling, translating, and rotating them to a consensus configuration. This procedure eliminated non-shape differences and generated size and shape variables. Wing size, represented by the centroid size (CS), was calculated as the square root of the sum of squared distances from each landmark to the centroid (Bookstein, 1991), serving as an isometric estimator of wing size for each species. Statistical comparisons of mean wing CS among species were performed using a non-parametric permutation test (1000 replicates) with Bonferroni correction. A significance threshold of *P* = 0.05 was applied, with values below this threshold considered significant.

To evaluate wing shape variability among species, 24 principal components (PCs) derived from wing shape variables were used as the final shape variables for discriminant analysis (DA), with results displayed on a factor map. Pairwise Mahalanobis distances were calculated to quantify shape differences between species. Statistical comparisons of wing shape, based on these pairwise Mahalanobis distances, were conducted using a non-parametric permutation test (1000 replicates) with Bonferroni correction, applying the same significance threshold of *P* = 0.05.

Validated classification (also known as leave-one-out classification) was performed to evaluate the accuracy of assigning each individual to its correct species, using the maximum likelihood method for size (Dujardin et al., 2017) and the Mahalanobis distance method for shape (Manly, 2004). This procedure accounted for prior probabilities to improve group classification accuracy by minimizing the influence of random variation (Klecka, 1980; McGarigal et al., 2000; Kovarovic et al., 2011). Adjusting for prior probabilities was particularly important in cases of imbalanced datasets, where unequal sample sizes across groups could bias classification outcomes. Incorporating prior probabilities enhanced classification accuracy by accounting for the uneven distribution of sample sizes (Chotelersak et al., 2024).

### 2.7 Outline-based GM analysis

For the outline-based GM method, Procrustes superimposition was conducted using Elliptic Fourier Analysis (EFA) to extract size and shape variables (Kuhl and Giardina, 1982). The contour size of the first submarginal cell was estimated by measuring its perimeter (size variable). To evaluate variability in contour shape among species, 23 PCs, derived from normalized elliptic Fourier coefficients, were used as final shape variables in DA. The results were visualized on a factor map, with pairwise Mahalanobis distances calculated to quantify contour shape differences between species. Statistical comparisons of size and shape variables among species were performed using a non-parametric permutation test (1000 replicates) with Bonferroni correction, maintaining a significance threshold of *P* = 0.05. Validated classification with adjusted prior probabilities was employed to enhance classification accuracy for size and shape derived from the first submarginal cell.

## 3 Results

**Fig. 3:**
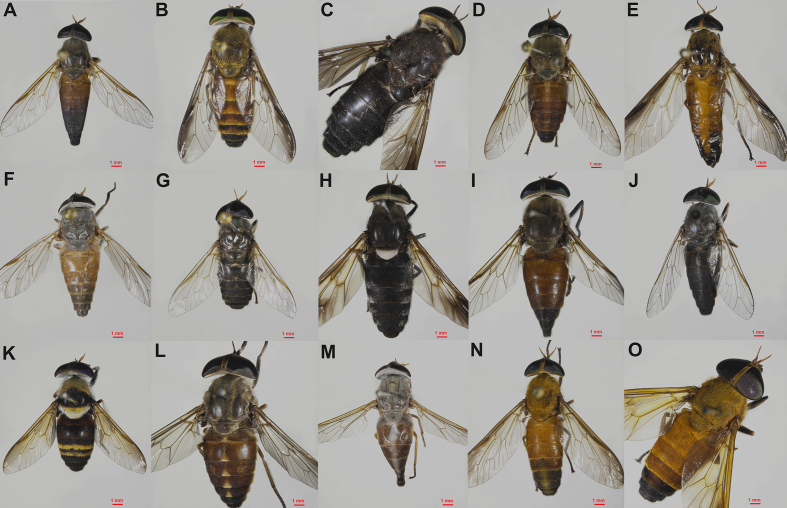
Images of the 15 *Tabanus* species analyzed in this study: *T. agnoscibilis* (**A**); *T. anabates* (**B**); *T. birmanicus* (**C**); *T. diversifrons* (**D**); *T. helvinus* (**E**); *T. konis* (**F**); *T. minimus* (**G**); *T. oknos* (**H**); *T. oxybeles* (**I**); *T. pugiunculus* (**J**); *T. rhinargus* (**K**); *T. rubicundus* (**L**); *T. systenus* (**M**); *T. tamthaiorum* (**N**); *T. thurmani* (**O**).

During the study period (March to July 2024), a total of 15 *Tabanus* species were collected from the study sites: *T. agnoscibilis*, *T. anabates*, *T. birmanicus*, *T. diversifrons*, *T. helvinus*, *T. konis*, *T. minimus*, *T. oknos*, *T. oxybeles*, *T. pugiunculus*, *T. rhinargus*, *T. rubicundus*, *T. systenus*, *T. tamthaiorum*, and *T. thurmani* (Fig. 3). Species confirmation was achieved through DNA barcoding, and GM analyses were conducted to evaluate morphological differences among species based on wing characteristics.

### 3.1 DNA barcoding

Thirty sequences of the *cox*1 gene, representing 15 *Tabanus* spp. (two specimens per species), were successfully amplified and sequenced. These sequences were compared against the GenBank and BOLD databases for species verification. The average nucleotide composition of the *cox*1 sequences was as follows: adenine (A) = 29.4%; thymine (T) = 39.5%; cytosine (C) = 14.9%; and guanine (G) = 16.2%. The AT content (68.9%) was notably higher than the GC content (31.1%), consistent with typical mitochondrial DNA patterns in insects.

The results of comparing *cox*1 sequences from this study with publicly available data in GenBank and BOLD databases are presented in Table 2. The *cox*1 sequences generated in this study for various species of *Tabanus* were largely consistent with the sequence data available in both databases. Most *Tabanus* species exhibited high sequence similarity, ranging from 96.20% to 100%, indicating strong matches with existing records. However, *T. helvinus* was identified as *T. aurilineatus*, and *T. minimus* matched sequences labeled as both *T. minimus* and *T. mesogaeus*. Additionally, two sequences of *T. tamthaiorum* did not match any existing sequences in GenBank but showed perfect matches with sequences in the BOLD database.

**Table 2:** Percentage sequence similarity for *Tabanus* spp. cytochrome *c* oxidase subunit 1 (*cox*1) sequences generated in this study, compared with reference sequences from the GenBank and BOLD databases.

ID	Species	GenBank ID	GenBank		BOLD	
			Species match	% Similarity (*n*)	Species match	% Similarity (*n*)
1	*T. agnoscibilis*	PQ462475	*T. agnoscibilis*	97.87–100 (3)	*T. agnoscibilis*	97.87–100 (3)
2	*T. agnoscibilis*	PQ462476	*T. agnoscibilis*	97.87–100 (3)	*T. agnoscibilis*	97.87–100 (3)
3	*T. anabates*	PQ462477	*T. anabates*	99.85–100 (2)	*T. anabates*	99.85–100 (2)
4	*T. anabates*	PQ462478	*T. anabates*	99.85–100 (2)	*T. anabates*	99.85–100 (2)
5	*T. birmanicus*	PQ462479	*T. birmanicus*	96.20–96.66 (3)	*T. birmanicus*	96.20–96.66 (3)
6	*T. birmanicus*	PQ462480	*T. birmanicus*	98.02–99.85 (3)	*T. birmanicus*	98.02–99.85 (3)
7	*T. diversifrons*	PQ462481	*T. diversifrons*	99.85 (1)	*T. diversifrons*	99.85 (1)
8	*T. diversifrons*	PQ462482	*T. diversifrons*	99.85 (1)	*T. diversifrons*	99.85 (1)
9	*T. helvinus*	PQ462483	*T. aurilineatus*	98.18–98.48 (3)	*T. aurilineatus*	98.18–98.48 (3)
10	*T. helvinus*	PQ462484	*T. aurilineatus*	98.18–98.48 (3)	*T. aurilineatus*	98.18–98.48 (3)
11	*T. konis*	PQ462485	*T. konis*	99.54 (1)	*T. konis*	99.54 (1)
12	*T. konis*	PQ462486	*T. konis*	99.39 (1)	*T. konis*	99.39 (1)
13	*T. minimus*	PQ462487	*T. minimus*	96.50–97.87 (3)	*T. minimus*	96.48–97.87 (5)
			*T. mesogaeus*	98.63–99.85 (3)	*T. mesogaeus*	98.63–99.85 (3)
14	*T. minimus*	PQ462488	*T. minimus*	96.50–97.87 (3)	*T. minimus*	96.48–97.87 (5)
			*T. mesogaeus*	98.63–99.85 (3)	*T. mesogaeus*	98.63–99.85 (3)
15	*T. oknos*	PQ462489	*T. oknos*	99.54–99.85 (3)	*T. oknos*	99.54–99.85 (3)
16	*T. oknos*	PQ462490	*T. oknos*	99.54–99.85 (3)	*T. oknos*	99.54–99.85 (3)
17	*T. oxybeles*	PQ462491	*T. oxybeles*	99.54–99.70 (2)	*T. oxybeles*	99.54–99.70 (2)
18	*T. oxybeles*	PQ462492	*T. oxybeles*	99.24–99.39 (2)	*T. oxybeles*	99.24–99.39 (2)
19	*T. pugiunculus*	PQ462493	*T. pugiunculus*	99.39–100 (6)	*T. pugiunculus*	99.39–100 (6)
20	*T. pugiunculus*	PQ462494	*T. pugiunculus*	99.24–99.85 (6)	*T. pugiunculus*	99.24–99.85 (6)
21	*T. rhinargus*	PQ462495	*T. rhinargus*	99.09–99.39 (3)	*T. rhinargus*	99.09–99.39 (3)
22	*T. rhinargus*	PQ462496	*T. rhinargus*	99.09–99.39 (3)	*T. rhinargus*	99.09–99.39 (3)
23	*T. rubicundus*	PQ462497	*T*. *rubicundus*	98.78–100 (3)	*T*. *rubicundus*	98.63–100 (50)
24	*T. rubicundus*	PQ462498	*T*. *rubicundus*	98.78–100 (3)	*T*. *rubicundus*	98.63–100 (50)
25	*T. systenus*	PQ462499	*T*. *systenus*	99.54 (2)	*T*. *systenus*	99.54 (2)
26	*T. systenus*	PQ462500	*T*. *systenus*	99.54 (2)	*T*. *systenus*	99.54 (2)
27	*T. tamthaiorum*	PQ462501	No match	No match	*T. tamthaiorum*	100 (1)
28	*T. tamthaiorum*	PQ462502	No match	No match	*T. tamthaiorum*	100 (1)
29	*T. thurmani*	PQ462503	*T. thurmani*	99.70–100 (2)	*T. thurmani*	99.70–100 (2)
30	*T. thurmani*	PQ462504	*T. thurmani*	99.54 (2)	*T. thurmani*	99.54 (2)

*Note*: All comparisons had 100% query coverage, indicating that our sequences span the entire length of the reference sequences in databases.

*Abbreviations*: *n*, number of sequences.

**Fig. 4:**
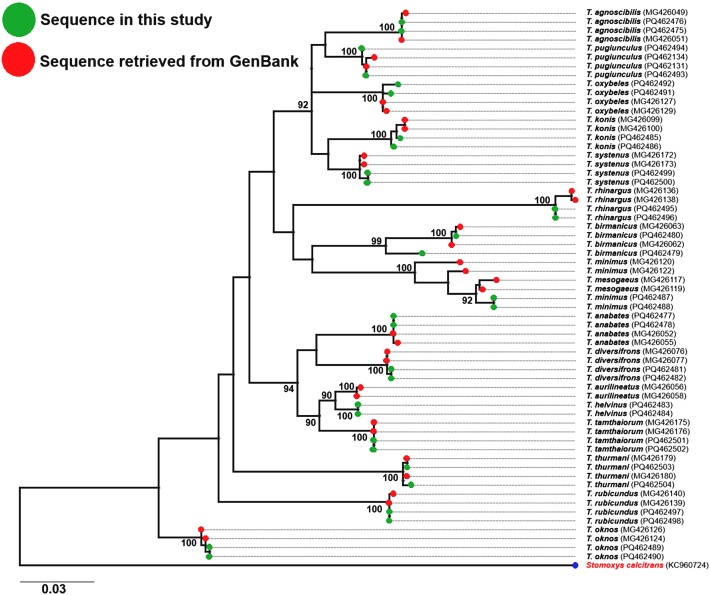
Maximum likelihood (ML) phylogenetic tree based on 62 *cox*1 sequences of 15 *Tabanus* species, including 30 sequences from this study (*green*) and 32 from GenBank (*red*). *Stomoxys calcitrans* served as the outgroup. Bootstrap values ≥ 90% are shown for branch support.

Phylogenetic relationships among *Tabanus* species, along with *S. calcitrans* as the outgroup, are illustrated in Fig. 4. ML analysis revealed distinct clusters for most species, strongly supported by bootstrap values ranging from 90% to 100%. However, *T. minimus* and *T. mesogaeus* exhibited less clear differentiation, forming closely related clusters.

### 3.2 Geometric morphometrics

The digitization of landmarks and pseudolandmarks (points) was meticulously carried out and verified for precision. Repeatability tests for wing shape demonstrated high precision, with 98% for the landmark-based GM method and 97% for the outline-based GM method. The influence of wing size on shape variation (allometric effects) was quantified at 62.3% (*r*^*2*^) for the landmark-based GM analysis and 58.5% for the outline-based GM analysis. Although these effects were significant, they were retained in the analysis because size-related shape variation is informative and contributes to the process of species identification.

#### 3.2.1 Landmark-based GM method

**Fig. 5:**
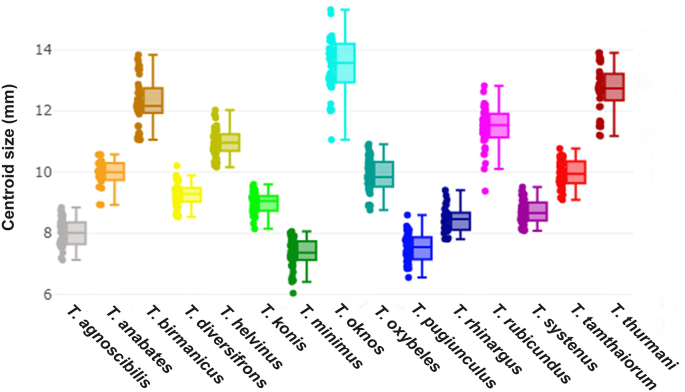
Boxplots illustrating wing centroid size variation among 15 *Tabanus* species. The horizontal line within each box represents the median, with the 25th and 75th quartiles shown. Colored dots represent individual specimens.

Wing CS variations among the 15 *Tabanus* species are summarized in Fig. 5. The mean wing CS ranged from 7.34 mm to 13.47 mm, with *T. oknos* exhibiting the largest wings and *T. minimus* the smallest. Significant differences in wing CS among species, as detailed in Table 3, confirm notable interspecies variation.

**Table 3:** Comparisons of wing centroid size (CS) among 15 *Tabanus* species based on landmark-based geometric morphometric analysis.

Species	*n*	Mean (mm)	Min-Max	Variance	SD
*T. agnoscibilis*	45	8.01 ^A, G^	7.13–8.84	0.19	0.44
*T. anabates*	25	9.96 ^B^	8.93–10.58	0.21	0.46
*T. birmanicus*	45	12.28 ^C^	11.06–13.83	0.60	0.78
*T. diversifrons*	45	9.27 ^B, E^	8.53–10.21	0.15	0.39
*T. helvinus*	45	10.98 ^D^	10.17–12.03	0.20	0.45
*T. konis*	45	8.99 ^E^	8.15–9.59	0.11	0.33
*T. minimus*	45	7.34 ^A^	6.04–8.07	0.23	0.48
*T. oknos*	43	13.47 ^F^	11.06–15.31	0.75	0.87
*T. oxybeles*	45	9.88 ^B^	8.75–10.91	0.31	0.56
*T. pugiunculus*	45	7.55 ^A, H^	6.56–8.60	0.21	0.46
*T. rhinargus*	29	8.45 ^E, G, H^	7.81–9.41	0.18	0.43
*T. rubicundus*	45	11.45 ^D^	9.38–12.82	0.42	0.65
*T. systenus*	45	8.70 ^E, G^	8.08–9.52	0.14	0.37
*T. tamthaiorum*	44	9.96 ^B^	9.10–10.77	0.19	0.43
*T. thurmani*	45	12.70 ^C, F^	11.18–13.90	0.57	0.75

*Note*: Different superscript letters indicate statistically significant differences (*P* < 0.05).

*Abbreviations*: *n*, sample size; Min, minimum; Max, maximum; SD, standard deviation.

**Fig. 6:**
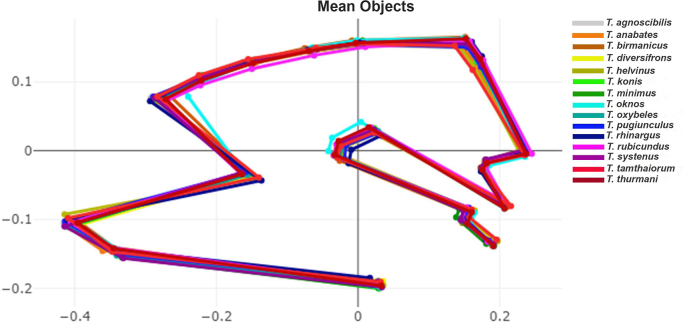
Superposed aligned mean configurations of wing landmarks among 15 *Tabanus* species from the landmark-based geometric morphometric analysis.

**Fig. 7:**
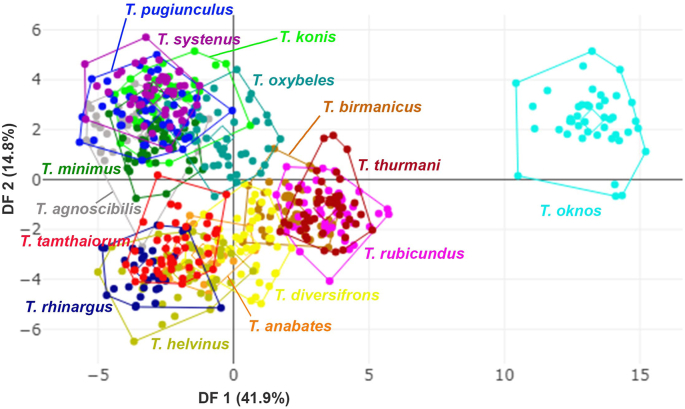
Factor map of the first two discriminant factors (DFs) among 15 *Tabanus* species based on landmark-based geometric morphometric analysis. Points represent individual specimens. The first discriminant factor (DF1) accounts for 41.9% of the variance, and DF2 accounts for 14.8% of the variance.

The superposition of mean wing shapes, shown in Fig. 6, highlights clear distinctions, particularly between *T. oknos* and other species. The DA of the first two discriminant factors (DF1 = 41.9%, DF2 = 14.8%) accounted for 56.7% of the total shape variation, as visualized in Fig. 7. The factor map illustrates significant shape variations among the species, with *T. oknos* demonstrating particularly distinct differences. Pairwise Mahalanobis distances, measuring interspecies shape differences, ranged from 3.8 (*T. agnoscibilis vs T. pugiunculus*) to 18.99 (*T. oknos* *vs* *T. rhinargus*). Table 4 presents statistically significant differences in wing shape among all species (*P* < 0.05).

**Table 4:** Mahalanobis distance matrix showing species-level differentiation among 15 *Tabanus* species based on landmark-based geometric morphometric analysis.

Species	1	2	3	4	5	6	7	8	9	10	11	12	13	14
1	–													
2	7.18	–												
3	9.01	8.41	–											
4	7.78	5.14	7.46	–										
5	7.76	5.96	9.81	7.40	–									
6	4.56	7.76	9.23	7.94	9.13	–								
7	6.02	8.48	8.65	8.29	9.42	7.09	–							
8	17.14	16.18	13.44	14.70	16.81	16.61	17.10	–						
9	5.37	6.74	7.32	6.20	8.23	5.63	6.49	14.37	–					
10	3.80	8.26	8.57	8.77	9.46	6.11	6.12	17.02	5.88	–				
11	8.99	9.19	9.40	9.15	10.06	10.69	9.31	18.99	9.56	8.85	–			
12	10.26	8.78	8.42	7.12	10.75	10.19	9.73	13.29	8.03	10.30	11.02	–		
13	4.45	8.42	9.62	7.70	8.69	4.59	6.08	16.80	5.25	6.08	10.99	10.57	–	
14	8.29	7.90	9.36	7.81	7.66	9.27	8.47	16.99	8.30	9.29	10.86	11.69	9.35	–
15	9.18	7.01	5.93	6.10	8.74	8.74	8.97	12.17	7.63	9.08	10.51	6.83	9.21	10.70

*Note*: The numbers in the table correspond to the following *Tabanus* species: 1, *T. agnoscibilis*; 2, *T. anabates*; 3, *T. birmanicus*; 4, *T. diversifrons*; 5, *T. helvinus*; 6, *T. konis*; 7, *T. minimus*; 8, *T. oknos*; 9, *T. oxybeles*; 10, *T. pugiunculus*; 11, *T. rhinargus*; 12, *T. rubicundus*; 13, *T. systenus*; 14, *T. tamthaiorum*; and 15, *T. thurmani*. Statistical analysis revealed that all pairwise comparisons were significantly different (*P* < 0.05).

Classification based on wing size showed low accuracy, with a validated classification score of 32.08% and an adjusted score for prior probabilities of 27% (Table 5). In contrast, classification based on wing shape yielded significantly higher accuracy, achieving a validated classification score of 97.33% and an adjusted score of 97%. Perfect classification accuracy (100%) was achieved for nine species: *T. anabates*, *T. birmanicus*, *T. diversifrons*, *T. minimus*, *T. oknos*, *T. rhinargus*, *T. rubicundus*, *T. tamthaiorum*, and *T. thurmani.*

**Table 5:** Validated classification accuracy for wing size and shape among 15 *Tabanus* species based on landmark-based geometric morphometric analysis.

Species	Wing size		Wing shape	
	Assigned	Accuracy (%)	Assigned	Accuracy (%)
*T. agnoscibilis* (*n* = 45)	11	24.44	40	88.89
*T. anabates* (*n* = 25)	8	32.00	25	100
*T. birmanicus* (*n* = 45)	19	42.22	45	100
*T. diversifrons* (*n* = 45)	21	46.67	45	100
*T. helvinus* (*n* = 45)	20	44.44	44	97.78
*T. konis* (*n* = 45)	8	17.78	42	93.33
*T. minimus* (*n* = 45)	8	17.78	45	100
*T. oknos* (*n* = 43)	26	60.47	43	100
*T. oxybeles* (*n* = 45)	18	40.00	42	93.33
*T. pugiunculus* (*n* = 45)	12	26.67	41	91.11
*T. rhinargus* (*n* = 29)	4	13.79	29	100
*T. rubicundus* (*n* = 45)	18	40.00	45	100
*T. systenus* (*n* = 45)	11	24.44	44	97.78
*T. tamthaiorum* (*n* = 44)	2	4.55	44	100
*T. thurmani* (*n* = 45)	18	40.00	45	100
Total accuracy (*n* = 636)	204	32.08	619	97.33
Adjusted total accuracy		27		97

*Note*: Validated classification scores represent the proportion of individuals correctly categorized, demonstrating the accuracy of the classification. Adjusted total accuracy accounts for the score after considering correct assignments that might have occurred by chance. This adjustment provides a more precise measure of the classification’s effectiveness.

#### 3.2.2 Outline-based GM method

**Fig. 8:**
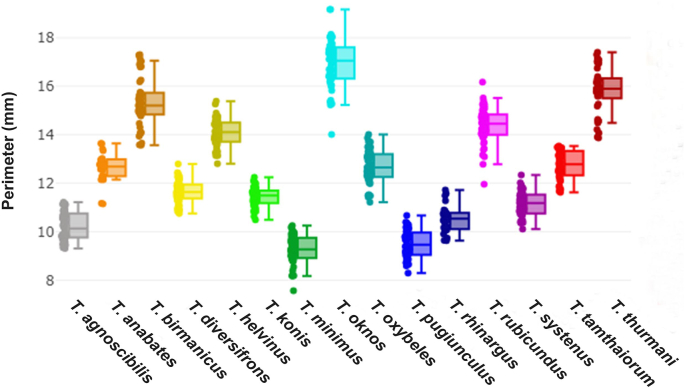
Boxplots illustrating wing cell perimeter variation among 15 *Tabanus* species. The horizontal line within each box represents the median, with the 25th and 75th quartiles shown. Colored dots represent individual specimens.

For the outline-based GM method, the perimeter of the first submarginal cell was used to represent size. Fig. 8 illustrates variations in wing cell perimeter among the species, with mean values ranging from 9.27 mm (*T. minimus*) to 16.92 mm (*T. oknos*). Significant differences in wing cell perimeter among species are detailed in Table 6.

**Table 6:** Comparisons of wing cell perimeter among 15 *Tabanus* species based on outline-based geometric morphometric analysis.

Species	*n*	Mean (mm)	Min-Max	Variance	SD
*T. agnoscibilis*	43	10.21 ^A, G^	9.31–11.22	0.33	0.57
*T. anabates*	24	12.64 ^B, F^	11.15–13.65	0.39	0.63
*T. birmanicus*	45	15.31 ^C, H^	13.57–17.31	1.07	1.03
*T. diversifrons*	45	11.67 ^B, K^	10.76–12.80	0.22	0.47
*T. helvinus*	45	14.10 ^D^	12.80–15.38	0.32	0.57
*T. konis*	45	11.44 ^B, I^	10.48–12.25	0.16	0.40
*T. minimus*	45	9.27 ^A^	7.56–10.25	0.35	0.59
*T. oknos*	44	16.92 ^E^	14.01–19.17	1.04	1.02
*T. oxybeles*	45	12.68 ^F^	11.21–14.01	0.48	0.69
*T. pugiunculus*	45	9.52 ^A, J^	8.29–10.67	0.32	0.56
*T. rhinargus*	28	10.52 ^G, I, J, K^	9.62–11.72	0.31	0.56
*T. rubicundus*	44	14.36 ^C, D^	11.95–16.18	0.61	0.78
*T. systenus*	45	11.16 ^G, I, K^	10.11–12.34	0.26	0.51
*T. tamthaiorum*	45	12.77 ^F^	11.62–13.53	0.32	0.57
*T. thurmani*	45	15.84 ^H^	13.86–17.41	0.89	0.95

*Note*: Different superscript letters indicate statistically significant differences (*P* < 0.05).

*Abbreviations*: *n*, sample size; Min, minimum; Max, maximum; SD, standard deviation.

**Fig. 9:**
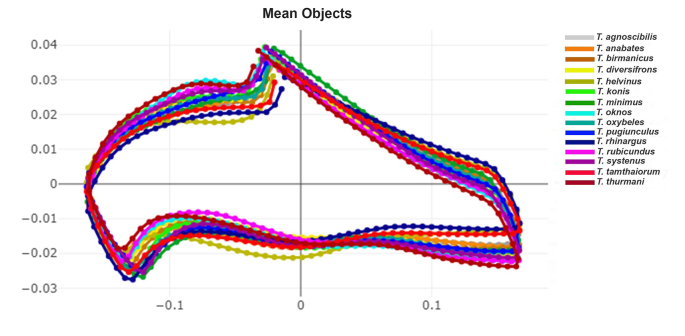
Superposed mean contours of the first submarginal cell among 15 *Tabanus* species from the outline-based geometric morphometric analysis.

**Fig. 10:**
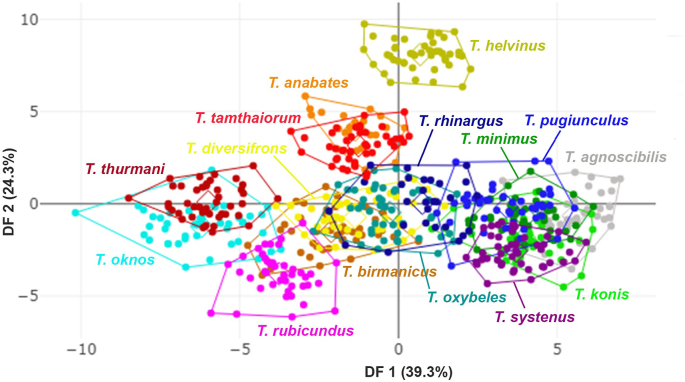
Factor map of the first two discriminant factors (DFs) among 15 *Tabanus* species based on outline-based geometric morphometric analysis. Points represent individual specimens. The x-axis represents the first discriminant factor (DF1), accounting for 39.3% of the variance, and the y-axis represents the second discriminant factor (DF2), which accounts for 24.3% of the variance.

The superimposed mean contours of the first submarginal cell among *Tabanus* species revealed species-specific differences, particularly in the curvature of this wing cell, as depicted in Fig. 9. In the DA, the first two discriminant factors (DF1 and DF2) accounted for 63.6% of the total shape variation, with DF1 contributing 39.3% and DF2 24.3% (Fig. 10). The factor map based on DF1 and DF2 highlighted distinct shape variations, with *T. helvinus* clearly separated from other species, showing no overlap. Pairwise Mahalanobis distances between species ranged from 4.28 (*T. birmanicus vs T. diversifrons*) to 12.94 (*T. helvinus vs T. rubicundus*). Significant differences in wing cell shape, as indicated by Mahalanobis distances, are presented in Table 7 (*P* < 0.05).

**Table 7:** Mahalanobis distance matrix showing species-level differentiation among 15 *Tabanus* species based on outline-based geometric morphometric analysis.

Species	1	2	3	4	5	6	7	8	9	10	11	12	13	14
1	–													
2	9.48	–												
3	8.81	8.42	–											
4	7.83	6.76	4.28	–										
5	10.65	7.35	10.88	10.22	–									
6	4.46	10.03	7.76	7.09	10.91	–								
7	5.78	8.75	8.06	7.73	10.69	6.04	–							
8	12.53	8.91	7.45	7.87	12.36	11.38	11.43	–						
9	7.91	7.52	5.24	4.98	9.94	6.31	5.65	7.73	–					
10	5.26	7.72	8.47	7.41	10.42	6.81	5.13	10.34	6.67	–				
11	7.00	9.00	7.19	6.35	10.57	7.87	8.57	10.24	7.38	8.11	–			
12	10.30	9.57	5.51	6.17	12.94	9.25	9.79	6.17	7.58	9.86	8.73	–		
13	4.95	9.61	7.94	7.76	11.68	4.57	5.09	10.48	6.46	4.92	8.25	8.85	–	
14	8.87	6.00	6.52	6.48	7.99	9.25	8.16	8.34	5.58	7.67	6.96	9.47	8.86	–
15	12.19	7.67	6.78	6.57	11.31	10.88	10.91	5.08	7.25	10.52	9.63	6.79	10.67	7.40

*Note*: The numbers in the table correspond to the following *Tabanus* species: 1, *T. agnoscibilis*; 2, *T. anabates*; 3, *T. birmanicus*; 4, *T. diversifrons*; 5, *T. helvinus*; 6, *T. konis*; 7, *T. minimus*; 8, *T. oknos*; 9, *T. oxybeles*; 10, *T. pugiunculus*; 11, *T. rhinargus*; 12, *T. rubicundus*; 13, *T. systenus*; 14, *T. tamthaiorum*; and 15, *T. thurmani*. Statistical analysis revealed that all pairwise comparisons were significantly different (*P* < 0.05).

Classification based on wing cell size yielded a low validated classification score of 28.12%, with an adjusted score for prior probabilities of 23% (Table 8). Conversely, classification based on wing cell shape achieved significantly higher accuracy, with a validated classification score of 96.37% and an adjusted score of 96%. Species with the highest classification accuracy (100%) included *T. anabates*, *T. helvinus*, *T. rubicundus*, and *T. tamthaiorum*, while the lowest accuracy was observed for *T. agnoscibilis* (88.37%).

**Table 8:** Validated classification accuracy for the size and shape of the first submarginal cell among 15 *Tabanus* species based on outline-based geometric morphometric analysis.

Species	Wing cell size		Wing cell shape	
	Assigned	Accuracy (%)	Assigned	Accuracy (%)
*T. agnoscibilis* (*n* = 43)	14	32.56	38	88.37
*T. anabates* (*n* = 24)	1	4.17	24	100
*T. birmanicus* (*n* = 45)	9	20.00	44	97.78
*T. diversifrons* (*n* = 45)	18	40.00	44	97.78
*T. helvinus* (*n* = 45)	5	11.11	45	100
*T. konis* (*n* = 45)	12	26.67	44	97.78
*T. minimus* (*n* = 45)	17	37.78	44	97.78
*T. oknos* (*n* = 44)	30	68.18	41	93.18
*T. oxybeles* (*n* = 45)	11	24.44	42	93.33
*T. pugiunculus* (*n* = 45)	6	13.33	44	97.78
*T. rhinargus* (*n* = 28)	4	14.29	25	89.29
*T. rubicundus* (*n* = 44)	11	25.00	44	100
*T. systenus* (*n* = 45)	14	31.11	43	95.56
*T. tamthaiorum* (*n* = 45)	10	22.22	45	100
*T. thurmani* (*n* = 45)	16	35.56	43	95.56
Total accuracy (*n* = 633)	178	28.12	610	96.37
Adjusted total accuracy		23		96

*Note*: Validated classification scores represent the proportion of individuals correctly categorized, demonstrating the accuracy of the classification. Adjusted total accuracy accounts for the score after considering correct assignments that might have occurred by chance. This adjustment provides a more precise measure of the classification’s effectiveness.

## 4 Discussion

The identification of horse flies is challenging due to their high morphological variability, often influenced by environmental factors. Variations in features such as the coloration of frontal calli, antennae, notopleural lobes, legs, and abdomen frequently result in misidentification (Krčmar et al., 2022). These challenges are exacerbated when specimens are aged or damaged, particularly when critical structures like antennae, legs, palpi, hairs, and markings are missing, increasing the likelihood of errors. This study introduced DNA barcoding and GM techniques as alternative methods for enhancing species classification, particularly for surveillance efforts in Western Thailand. These methods also serve as a guideline for application in other regions. In addition to overcoming the limitations of morphological methods, applying these advanced techniques in biodiverse regions such as Kanchanaburi offers a unique opportunity to test their effectiveness across diverse ecological settings. The collection of 15 horse fly species from Kanchanaburi Province aligns with previous surveys that identified this region as a biodiversity hotspot for horse flies (Changbunjong et al., 2018b). The observed diversity is influenced by environmental factors such as prey animal density and local climate, with forested areas hosting a greater variety of species compared to open grasslands (Lucas et al., 2020). Discrepancies in the number of species identified in this study may not match those reported in earlier studies, possibly due to differences in the timing of collections. To address the challenges posed by high morphological variability and enhance species identification in such biodiversity regions, DNA barcoding was employed as a reliable molecular tool.

DNA barcoding is a powerful taxonomic tool for identifying species using a standardized short DNA sequence (Antil et al., 2023). The sequence from an unknown sample is compared to a reference library for identification (Kress et al., 2015). In this study, DNA barcoding confirmed the true species of horse flies before GM analyses. Species identification based on comparisons with GenBank and BOLD database sequences generally corresponded with morphological identifications. For mismatches, *T. helvinus* was identified as *T. aurilineatus*, likely due to incorrect sequence records in public databases. Errors in DNA barcoding-based insect identification often arise from human factors such as specimen misidentification, sample confusion, and contamination (Cheng et al., 2023). The horse flies *T. helvinus* and *T. aurilineatus* have very similar morphological traits, complicating their distinction. However, *T. helvinus* is identifiable by its golden-orange-yellow pale abdominal hairs and darker legs, particularly the forelegs. Conversely, *T. aurilineatus* is confined to peninsular Thailand, with no records north of the Isthmus of Kra in Prachuap Khiri Khan Province, Western Thailand (Burton, 1978).

Sequence data for *T. minimus* in this study matched sequences identified as both *T. minimus* and *T. mesogaeus*. This finding aligns with prior research in Thailand, which indicated that the *cox*1 gene marker cannot reliably differentiate between *T. minimus* and *T. mesogaeus*, requiring additional molecular markers for precise identification (Changbunjong et al., 2018a). While DNA barcoding successfully identified most species, certain closely related species posed challenges, underscoring the need for additional molecular markers and complementary approaches. Despite genetic similarity, the two species exhibit distinct morphological differences. *Tabanus minimus* is identifiable by its fully tomentose frontoclypeus, white beard, and other whitish hairs on the face, thorax, and abdomen, along with a hyaline wing with a clear costal cell (Burton, 1978). *Tabanus mesogaeus*, on the other hand, features a completely tomentose frontoclypeus, a black beard, and typically lacks pale hairs on the thorax and abdomen, paired with a hyaline wing (Burton, 1978). In this study, all specimens identified as *T. minimus* were distinctly recognized by their beard color.

Sequence data in the BOLD database are crucial for confirming the identity of unknown specimens, which is essential for accurate insect taxonomy and systematic studies (Krčmar et al., 2022). In this study, we also compared horse fly sequences with those in the GenBank genetic database. Notably, the identification results from BOLD helped resolve gaps that BLAST searches in GenBank could not, particularly for *T. tamthaiorum*, whose sequence data lacked a match in GenBank. Beyond resolving gaps in sequence data, constructing a phylogenetic tree from DNA barcoding sequences further validated the species identifications and highlighted evolutionary relationships.

The ML phylogenetic tree constructed from *cox*1 barcode sequences of 15 horse fly species validated species identifications and illustrated their phylogenetic relationships. Each species formed a distinct clade with strong bootstrap support exceeding 90%, confirming their morphological identifications. The findings underscore the efficacy of DNA barcoding as a species identification tool for horse flies, consistent with successful identifications in regions such as India (Banerjee et al., 2015), Croatia (Krčmar et al., 2022), and East Africa (Mugasa et al., 2018).

Surveillance of vector species by local agencies often faces budget constraints. This study employed the GM technique for classifying horse fly species, offering the advantage of low cost. GM has previously been shown to effectively classify diverse fly species such as blow flies (Sontigun et al., 2017; Limsopatham et al., 2022), flesh flies (Sontigun et al., 2019), and stomoxyine flies (Changbunjong et al., 2016). Insect wings, the focus of this technique, provide distinct species-specific characteristics and an almost two-dimensional structure that minimizes digitizing errors (Dujardin, 2008). Moreover, wing cells, the components of the wing, exhibit unique characteristics for each species (Weluwanarak et al., 2024). Our analysis using both landmark-based GM for the entire wing and outline-based GM for the first submarginal cell demonstrated that wing shape is a reliable metric for classifying horse fly species, achieving high adjusted total accuracy scores of 97% and 96%, respectively. The accuracy of the landmark-based GM method aligns with findings by Changbunjong et al. (2021), who reported a classification accuracy of 96.59% for closely related *Tabanus* species, including *T. megalops*, *T. rubidus*, and *T. striatus*. Furthermore, the classification accuracy based on the first submarginal cell in this study exceeded previous results, surpassing the 86.67% accuracy reported for *T. megalops*, *T. rubidus*, and *T. striatus* (Changbunjong et al., 2024a). These findings suggest that variations in wing cell structure between species may influence classification outcomes.

Analyses of wing size and wing cell size using both landmark- and outline-based GM methods demonstrated low accuracy in classifying horse fly species, with accuracy rates of 27% and 23%, respectively. Size has proven unreliable for species distinction due to its sensitivity to environmental fluctuations and overlap among species (Dujardin, 2008). The findings of the present study confirm that size is not an effective criterion for species classification in horse flies. While wing shape proved highly effective for species distinction, the size limitations as a classification criterion became evident due to its sensitivity to environmental variations.

The potential of wing shape and wing cell shape as discriminators for vector species is well-established (Dujardin, 2008; Kaba et al., 2017). In this study, despite some overlap in factor maps derived from DA, Mahalanobis distances revealed statistically significant differences between all species pairs. Most classifications achieved high correct identification rates in both landmark- and outline-based GM analyses, confirming the species-specific traits inherent in wing and wing cell shapes. However, classification results varied depending on the GM method used, reflecting the unique shape characteristics captured by each approach.

The landmark-based GM analysis distinctly identified *T. oknos* by its unique wing shape, characterized by the first posterior cell being closed before the hind margin, unlike other species with an open first posterior cell. This differentiation relied on anatomical landmarks across the wing, which the outline-based GM method focused solely on wing cells could not detect. Conversely, the outline-based GM method effectively highlighted distinctive features in *T. helvinus*, such as the prominently convex curves of the first submarginal cell, which the landmark-based method could not ascertain. While the landmark-based GM method is favored for its simplicity, the outline-based method is indispensable for examining species-specific wing cell shapes, providing critical insights for accurate classification (Changbunjong et al., 2024a). Based on our findings, both GM methods yielded similar classification accuracies. Therefore, when damaged wings prevent the use of the landmark-based GM method, the outline-based GM method serves as a viable alternative. These complementary GM methods not only enhance classification accuracy but also provide practical solutions for field conditions where specimens are damaged or incomplete, underscoring their broader utility.

## 5 Conclusions

This study presents effective alternative tools for classifying horse flies, offering significant advancements in vector monitoring and management efforts. DNA barcoding has proven highly effective, accurately identifying nearly 15 species of horse flies. However, caution is necessary regarding potentially misidentified sequences in databases, which should be thoroughly verified with each analysis. The landmark-based GM method, focusing on wing shape, and the outline-based GM method, targeting the first submarginal cell shape, both demonstrated high accuracy in classifying horse flies from Western Thailand. These findings highlight the value of an integrated approach to species classification. Initial morphological classification should be followed by a random selection of specimens for DNA barcoding to confirm species identity. Subsequently, the landmark-based GM method should be applied to verify all specimens. In cases where wings are damaged but the first submarginal cell remains intact, the outline-based GM method provides a reliable alternative. Employing these complementary methods will significantly enhance the efficiency and accuracy of horse fly species classification.

## CRediT authorship contribution statement

**Tanasak Changbunjong:** Conceptualization, Investigation, Methodology, Formal analysis, Data curation, Writing – original draft, Writing – review & editing, Visualization, Supervision, Project administration, Funding acquisition. **Thekhawet Weluwanarak:** Investigation, Methodology, Visualization, Formal analysis. **Sedthapong Laojun:** Investigation, Methodology, Formal analysis, Writing – original draft. **Tanawat Chaiphongpachara:** Conceptualization, Investigation, Methodology, Formal analysis, Data curation, Writing – original draft, Writing – review & editing, Visualization.

## Ethical approval

The study received approval from the Animal Care and Use Committee of the Faculty of Veterinary Science at Mahidol University, Thailand (Ref. MUVS-2023-10-70).

## Funding

This research was funded by 10.13039/501100004156Mahidol University (Fundamental Fund: fiscal year 2024 by National Science Research and Innovation Fund (NSRF)), grant number FF-132/2567.

## Declaration of competing interests

The authors declare that they have no known competing financial interests or personal relationships that could have appeared to influence the work reported in this paper.

## Data Availability

The data supporting the conclusions of this article are included within the article and its supplementary file. The newly generated sequences were submitted to the GenBank database under the accession numbers PQ462475-PQ462504.
