# Supplementary material for: Species classification of Tabanus (Diptera: Tabanidae) in Western Thailand: Integrating DNA barcoding and modern morphometrics
Source: Curr Res Parasitol Vector Borne Dis. 2025 Jan 13;7:100243. doi: 10.1016/j.crpvbd.2025.100243 (PMC11848769; doi:10.1016/j.crpvbd.2025.100243)
Supplement: Multimedia component 1 [file mmc1.pdf]

Supplementary Table S1. Sequence sources and GenBank accession numbers for *Tabanus* species and the outgroup used in the construction of the phylogenetic tree.

Species	*n*	Sequence source	GenBank accession number
Tabanus agnoscibilis	2	This study	PQ462475, PQ462476
Tabanus agnoscibilis	2	GenBank	MG426049, MG426051
Tabanus anabates	2	This study	PQ462477, PQ462478
Tabanus anabates	2	GenBank	MG426052, MG426055
Tabanus birmanicus	2	This study	PQ462479, PQ462480
Tabanus birmanicus	2	GenBank	MG426062, MG426063
Tabanus diversifrons	2	This study	PQ462481, PQ462482
Tabanus diversifrons	2	GenBank	MG426076, MG426077
Tabanus helvinus	2	This study	PQ462483, PQ462484
Tabanus aurilineatus	2	GenBank	MG426056, MG426058
Tabanus konis	2	This study	PQ462485, PQ462486
Tabanus konis	2	GenBank	MG426099, MG426100
Tabanus minimus	2	This study	PQ462487, PQ462488
Tabanus minimus	2	GenBank	MG426120, MG426122
Tabanus mesogaeus	2	GenBank	MG426117, MG426119
Tabanus oknos	2	This study	PQ462489, PQ462490
Tabanus oknos	2	GenBank	MG426124, MG426126
Tabanus oxybeles	2	This study	PQ462491, PQ462492
Tabanus oxybeles	2	GenBank	MG426127, MG426129
Tabanus pugiunculus	2	This study	PQ462493, PQ462494
Tabanus pugiunculus	2	GenBank	MG426131, MG426134
Tabanus rhinargus	2	This study	PQ462495, PQ462496
Tabanus rhinargus	2	GenBank	MG426136, MG426138
Tabanus rubicundus	2	This study	PQ462497, PQ462498
Tabanus rubicundus	2	GenBank	MG426139, MG426140
Tabanus systenus	2	This study	PQ462499, PQ462500
Tabanus systenus	2	GenBank	MG426172, MG426173
Tabanus tamthaiorum	2	This study	PQ462501, PQ462502
Tabanus tamthaiorum	2	GenBank	MG426175, MG426176
Tabanus thurmani	2	This study	PQ462503, PQ462504
Tabanus thurmani	2	GenBank	MG426179, MG426180
Stomoxys calcitrans	1	GenBank	KC960724
